# Long term effectiveness of once-daily unboosted atazanavir plus abacavir/lamivudine as a switch strategy in subjects with virological suppression

**DOI:** 10.7448/IAS.17.4.19810

**Published:** 2014-11-02

**Authors:** Josep M Llibre, Alessandro Cozzi-Lepri, Jorge Antonio Valencia La Rosa, Court Pedersen, Matti Ristola, Marcelo Losso, Amanda Mocroft, Victor M. Mitsura, Vidar Ormaasen, Fernando Maltez, Marek Beniowski, Roger Paredes, on behalf of Eurosida in Eurocoord

**Affiliations:** 1 HIV Unit, Hospital Germans Trias i Pujol/ Lluita contra la SIDA Fndn., Barcelona, Spain; 2 Research Department of Infection & Population Health, University College London, London, UK; 3 HIV Unit, Hospital Germans Trias i Pujol, Barcelona, Spain; 4 Department of Infectious Diseases, Odense University Hospital, Odense, Denmark; 5 Infectious Diseases, Helsinki University Central Hospital, Helsinki, Finland; 6 Infectious Diseases, Hospital General de Agudos J. M. Ramos Mejía, Buenos Aires, Argentina; 7 Epidemiology and Medical Statistics, University College, London, UK; 8 Infectious Diseases, Gomel State Medical University, Gomel, Belarus; 9 Infectious Diseases, Ullevål University, Oslo, Norway; 10 Infectious Diseases, Curry Cabral, Lisbon, Portugal; 11 Diagnostics and Therapy for AIDS, Specialistic Hospital, Chorzów, Poland; 12 IrsiCaixa Institute, IrsiCaixa Fndn, Barcelona, Spain; 13 Epidemiology and Infectious Diseases, CPHIV, Copenhagen, Denmark

## Abstract

**Background:**

Use of unboosted atazanavir (ATV_400_) is approved in the US but not in Europe [1]. Due to pharmacokinetic interactions it should not be used with tenofovir but can be used with abacavir/lamivudine (ABC/3TC) [1, 2,3]. Effectiveness data of ATV_400_+ABC/3TC as a switch strategy in clinical routine however are scant.

**Methods:**

We evaluated treatment outcomes of ATV_400_+ABC/3TC in pre-treated subjects in the EuroSIDA cohort with undetectable HIV-1 RNA, and previous ABC experience or assumed previous HLA B57*01 testing. We performed a time to loss of virologic response (TLOVR below 50 c/mL) and a snapshot analysis at 48, 96 and 144 weeks. Virological failure (VF) was defined as a confirmed plasma HIV-1 RNA >50 c/mL.

**Results:**

We included 258 subjects: 176 (68%) male, median age 46 (IQR 41, 53) y, 225 (87.2%) white, hepatitis virus co-infection 36%, median baseline CD4 at switch 540 cells (360, 700), time with VL≤ 50 c/mL 45 (24, 69) months. The median calendar year of switching was 2008 (2006, 2010). The 3rd drug in previous regimen was ATV/r in 70 (27.1%), other PI/r in 25 (9.7%), and other 163 (63.2%); 85 (32.9%) had previously failed with a PI. The virological response at 48/96/144 weeks was, respectively, 89.5 [95% CI 85.1, 92.9]/88 [83.4, 91.7]/86.3% [81.6, 90.4] (TLOVR, composite endpoint failure or stop for any reason) and the risk of VF was 8.3/7.6/7.6%. In the snapshot analysis HIV-RNA was below 50 c/mL in 72.5/65.9/51.6%, respectively, and >50 c/mL in 6.6/5.4/4.3%. Only 0.8/1.9/3.5% discontinued due to adverse events. There was a high rate of discontinuations due to other reasons or with VL missing in window. In a multivariate adjusted analysis, we observed an association between VF and nadir CD4 count (RH 0.60 [0.39, 0.93] per 100 cells higher), time with VL≤50 c/mL (RH 0.89 [0.81, 0.98] per 6 months longer) and previous failure with a PI (3.04 [1.36, 6.80]). There was no association with gender, age, hepatitis virus co-infection, CD4 count at time of switching or third drug used in the previous regimen.

**Conclusions:**

A switch to ATV_400_+ABC/3TC in selected subjects with HIV-RNA below 50 c/mL is associated with relatively low rates of VF and discontinuation due to adverse events. Use might be considered in those with long-term suppression and without prior PI failure. Larger cohorts are required to further define the appropriate selection criteria.

**Figure 1:**
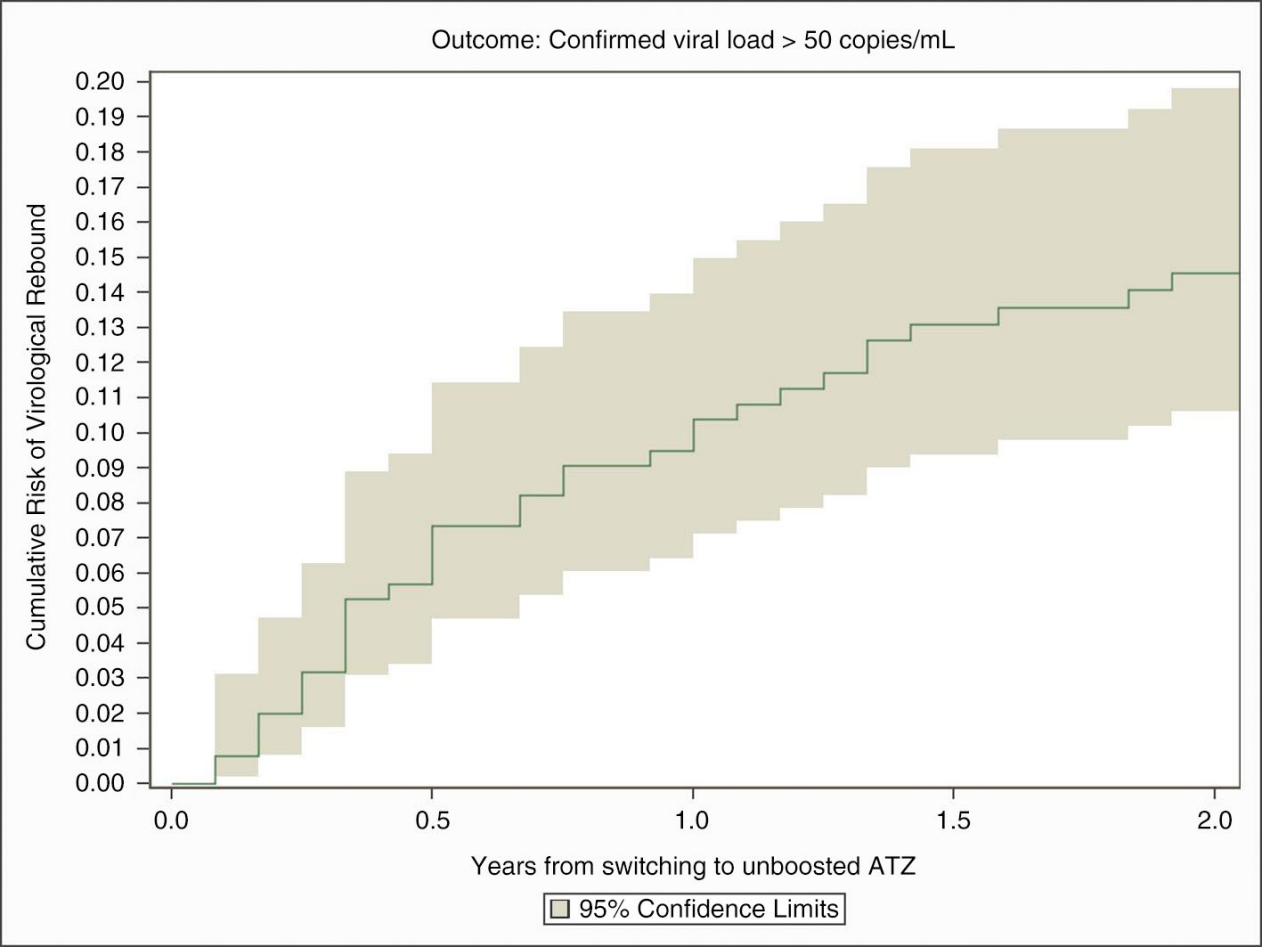
Cumulative Risk of loss of virological suppression.
